# Primary closure for pancreatic duct after stenting assisted by multiple endoscopes can be a new surgical method for the treatment of main pancreatic duct stones associated with pancreatic duct dilation

**DOI:** 10.1093/gastro/goae041

**Published:** 2024-04-30

**Authors:** Dongyao Xu, Linpei Wang, Wei Wang

**Affiliations:** Department of Hepatobiliary and Pancreatic Surgery, The Second Affiliated Hospital of Fujian Medical University, Quanzhou, Fujian, P. R. China; Department of Hepatobiliary and Pancreatic Surgery, The Second Clinical Medical College of Fujian Medical University, Fujian, P. R. China; Department of Hepatobiliary and Pancreatic Surgery, The Second Affiliated Hospital of Fujian Medical University, Quanzhou, Fujian, P. R. China; Department of Hepatobiliary and Pancreatic Surgery, The Second Clinical Medical College of Fujian Medical University, Fujian, P. R. China; Department of Hepatobiliary and Pancreatic Surgery, The Second Affiliated Hospital of Fujian Medical University, Quanzhou, Fujian, P. R. China; Department of Hepatobiliary and Pancreatic Surgery, The Second Clinical Medical College of Fujian Medical University, Fujian, P. R. China

## Introduction

Pancreatic duct stones typically manifest in patients with chronic pancreatitis and stem from diverse etiologies. Notably, they cannot be cured through non-surgical treatment and generally require surgical intervention. Conventional treatment methods for pancreatic duct stones include endoscopic retrograde cholangiopancreatography (ERCP), lithotripsy, and surgical intervention. ERCP requires highly trained and experienced doctors to perform due to its complexity and risks. There is still a risk of stone recurrence after pancreatic duct stone lithotripsy, and further treatment may be needed. And due to the overall health status of patients or other factors, not all patients are suitable for pancreatic duct stone lithotripsy. Surgical approaches frequently alter the gastrointestinal anatomy of the patients and usually promote pancreatic inflammation [1]. Herein, a novel method termed “primary closure for pancreatic duct after stenting assisted by multiple endoscopes” was pioneered to address the drawbacks associated with current surgical approaches. This innovative technique leveraged the advantages of several minimally invasive treatment techniques, including laparoscopy, cholangioscopy, Holmium laser lithotripsy, and duodenoscopy. To the best of our knowledge, this is the first study to devise a strategy encompassing multiple minimally treatment approaches. Moreover, this procedure was successfully implemented in three patients diagnosed with chronic pancreatitis featuring main pancreatic duct stones, all of whom achieved favorable clinical outcomes. The following sections provide a comprehensive overview of the treatment process, utilizing a patient as a representative example. (The data of the other two patients can be viewed in the Supplementary Data.)

## Patient

**Figure 1.**
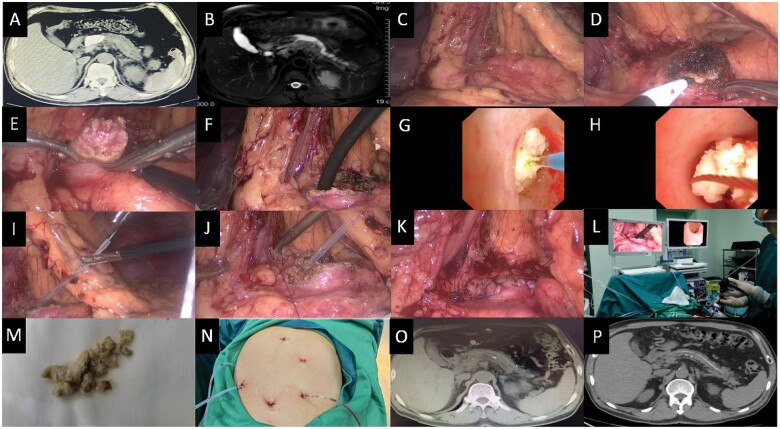
Image data of preoperative, intraoperative, and postoperative. (A) CT images of the pancreas prior to surgery illustrating pancreatic duct stones in the main pancreatic duct at the pancreatic neck. (B) MRI images of the pancreas before surgery displayed pancreatic duct stones in the main pancreatic duct at the pancreatic neck. (C) Dilated pancreatic duct. (D) Excising the main pancreatic duct with an electrocoagulation hook. (E) Extruding partial pancreatic duct stones. (F) Choledochoscopy exploration. (G) The Holmium laser was utilized to fragment stones in the main pancreatic duct under direct vision during choledochoscopy. (H) The choledochoscope was combined with a basket to extract the stones. (I) Pancreatic stents were inserted into pancreatic ducts. (J) and (K) Primary closure of the pancreatic duct. (L) Screening during surgery. (M) Pancreatic duct stones. (N) Postoperative incision. (O) CT images (1 month after surgery). (P) CT images (1 year after surgery).

The patient was a 57-year-old male with a past medical history of type 2 diabetes and hypertension. He was admitted to our department with abdominal pain for 1 month as the chief complaint. Intriguingly, the pain was not related to diet or body position and did not radiate to the back. The patient's hypertension was managed with amlodipine. Despite using insulin for glycemic control, it remained uncontrolled. There was no history of biliary or pancreatic duct stones before hospitalization. Likewise, the patient had no history of surgical interventions, smoking, or alcohol consumption. His BMI was 21.3 kg/m^2^. Magnetic resonance imaging and computed tomography (CT) displayed a dilated pancreatic duct (16 mm) with the presence of large stones (3.5 cm × 1.3 cm) in the pancreatic neck (Figure 1A and B). Therefore, a preoperative diagnosis of chronic pancreatitis with obstructive pancreatic duct stones was made. In March 2022, the patient underwent a surgical procedure that involved ‘laparoscopic pancreatic duct lithotomy + choledochoscopy + holmium laser lithotripsy + endoscopic pancreatic duct stent placement + primary closure for pancreatic duct’.

## Surgical procedure

Following anesthesia induction, the intervention was performed with the patient in the supine position. Briefly, an observation hole and another four operating holes were established. Next, the greater omentum was exposed. Then, the stomach was gently pulled upwards to expose the pancreas and pancreatic duct (Figure 1C). The dilated pancreatic duct in the pancreatic neck was incised approximately 3 cm laterally using a unipolar electric knife (Figure 1D). Following this, the pancreatic duct stones were extruded under direct vision. (Figure 1E). The pancreatic duct was subsequently explored using a 6-mm diameter choledochoscope (Figure 1F). Holmium laser lithotripsy (Figure 1G) and basket lithotomy (Figure 1H) were performed to extract the stones. Under choledochoscopic guidance, a zebra guide wire was inserted into the duodenal lumen through the duodenal papilla. Thereafter, an 8.5 Fr modified plastic stent was introduced into the pancreatic duct over the guidewire (Figure 1I). The pancreatic duct was then sutured using 4–0 PDS (Ethicon, Johnson, and Johnson Medical) (Figure 1J and K). Drainage tubes were placed at the pancreatic duct suture and Winslow hole, and the abdominal cavity was closed (Figure 1N).

## Results

The surgical procedure lasted for 140 minutes, and intraoperative blood loss was roughly 20 mL. As anticipated, the patient’s postoperative recovery was uneventful. Postoperative blood and drainage fluid amylase levels revealed no signs of pancreatic fluid leakage. The patient was discharged 10 days after the operation. One month after surgery, images of the follow-up CT scan depicted the successful clearance of the majority of stones, with the pancreatic duct stent in the proper position (Figure 1O). The patient did not suffer from abdominal pain or other symptoms. Besides, blood glucose levels remained within the normal range without the use of insulin. His weight increased compared to the baseline. The patient underwent a follow-up CT scan (Figure 1P) 1 year after the surgical procedure, revealing that the pancreatic duct stent maintained its position, with no further increase in the number of pancreatic duct stones.

## Discussion

Pancreatic duct stones can be managed via various treatment modalities, including non-surgical approaches, endoscopic interventions, and surgical procedures. The primary objective of surgical treatment is to alleviate pancreatic duct obstruction, preserve pancreatic duct patency, and ensure sufficient drainage of pancreatic juice [2, 3]. Traditional surgical methods include pancreatectomy and drainage. The duodenum-preserving pancreatic head resection (DPPHR) procedure is a modified version of the Beger operation that offers a less invasive alternative. However, the successful execution of DPPHR necessitates a high level of surgical skill and experience due to challenges in safeguarding the descending portion of the duodenum and the common bile duct, which may be susceptible to injury and ischemia. Another established procedure, namely the Partington-Rochelle operation, is frequently adopted as a drainage surgery. It has a satisfactory decompressive effect and is widely used in the clinical setting. However, the operation alters the anatomy of the patient’s digestive tract, which in turn influences digestive tract patency and increases the risk of postoperative ileus and inflammation of the pancreatic duct [1]. ERCP, extracorporeal shockwave lithotripsy (ESWL), and pancreatoscopy-directed lithotripsy are also minimally invasive methods for the treatment of pancreatolithiasis [4]. For larger stones, a combination of ESWL and pancreatoscopy-directed lithotripsy appears to be the optimal choice [3]. However, ERCP is challenging, requiring experienced endoscopic surgeons and carrying potentially severe complications. Herein, the novel method was pioneered to address the drawbacks associated with current surgical approaches. And in centers lacking experience in managing pancreatic duct stones with ERCP, pancreaticoduodenoscopy, or extracorporeal lithotripsy equipment, our method offers a more minimally invasive option.

We speculated that patients with a pancreatic duct diameter of 10 mm and stones primarily concentrated in the main pancreatic duct would be eligible for this novel method. Pancreatic ducts with a diameter exceeding 10 mm can be explored via choledochoscopy. Pancreatic ducts with a diameter exceeding 10 mm are not easily narrowed after primary closure. Considering that a choledochoscope cannot access the branch pancreatic duct for exploration, this method is suitable for patients with stones concentrated in the main pancreatic duct. Our approach employs a pancreatic duct stent to facilitate the drainage of pancreatic juice, attenuate pancreatic duct pressure, and prevent the formation of stones in the main pancreatic duct. On the one hand, it can minimize the risk of pancreatic fistulas. On the other hand, it can support the pancreatic duct and prevent pancreatic duct stenosis after primary closure. Compared to traditional resection and drainage techniques, this approach is simpler to execute. Additionally, our method eliminates the need for ionization of the duodenum and pancreas, thereby lowering the risk of injury to the duodenum and common bile duct. Furthermore, our approach minimizes the necessity for extensive removal of organs and tissues. The approach does not encompass pancreaticojejunostomy, which is in line with the physiological continuity of the digestive tract. The pancreatic duct stent can be removed using a duodenoscope after subsequent therapy [5]. It is worthwhile acknowledging that this new surgical technique demands extensive expertise in pancreatic duct suturing. Otherwise, inadequate pancreatic duct sutures may elevate the risk of pancreatic leakage, warranting long-term drainage.

The primary closure for the pancreatic duct after stent placement aligns with the concept of minimally invasive surgery for the treatment of main pancreatic duct stones associated with pancreatic duct dilation. The majority of stones were cleared in the three patients treated in our center using this method. Throughout the follow-up period, the patients remained asymptomatic without experiencing complications. Nevertheless, this method necessitates further exploration and adoption in the clinical setting.

## Supplementary Material

goae041_Supplementary_Data
