# Supplementary material for: Primary closure for pancreatic duct after stenting assisted by multiple endoscopes can be a new surgical method for the treatment of main pancreatic duct stones associated with pancreatic duct dilation
Source: Gastroenterol Rep (Oxf). 2024 Apr 30;12:goae041. doi: 10.1093/gastro/goae041 (PMC11060864; doi:10.1093/gastro/goae041)
Supplement: goae041_Supplementary_Data [file goae041_supplementary_data.docx]

**Appendix**

A total of three patients opted for the surgical approach of pancreatic duct incision and primary suture. Initially, due to lack of experience, one patient underwent open surgery while the other two underwent laparoscopic surgery. One patient has already been described in the manuscript. The following provides information about the remaining two patients.

Patient 1

Patient was a 39-year-old female who presented with one-day history of abdominal pain at our hospital. CT and MRCP examinations revealed dilatation (1.4cm) with calculi (1.2cm*1.6cm) in the main pancreatic duct along with pancreatic atrophy (**Figure** **1**). She had no prior medical history and her BMI was 22.1 kg/m². Open surgery was performed in February 2022, following a similar intraoperative procedure as described in the manuscript; choledochoscopy-guided removal of calculi under direct vision after incising the pancreatic duct, followed by insertion of a 8Fr pancreatic duct stent and primary suturing using PDS material were carried out successfully. Postoperative CT scan demonstrated significant reduction in calculi within the main pancreatic duct (**Figure** **2**). The patient was discharged ten days post-surgery. During telephonic follow-up conducted one year later, she reported absence of any recurrent abdominal pain symptoms. However, no further CT review was conducted.

Patient 2

The patient is a 37-year-old woman. She had no symptoms like abdominal pain. Physical examination revealed pancreatic duct stones. She has a history of surgery for breast fibroma. CT and MRCP in our hospital indicated that "pancreatic duct dilatation (1.0cm) was mainly confined to pancreatic duct calculi at the head of the pancreas (1.0cm*1.3cm) and partial calculi at the tail of the pancreas"(**Figure 3**). In June 2022, 'laparoscopic pancreatic duct lithotripsy + choledochoscopy+ holmium laser lithotripsy + endoscopic pancreatic duct stent placement + primary closure for pancreatic duct '. The postoperative recovery of the patient was very smooth. Postoperative CT showed that the head stone was basically removed (**Figure** **4**), but the tail stone was not removed. The patient was discharged 7 days after surgery. However, The patient refused to be re-examined after discharge


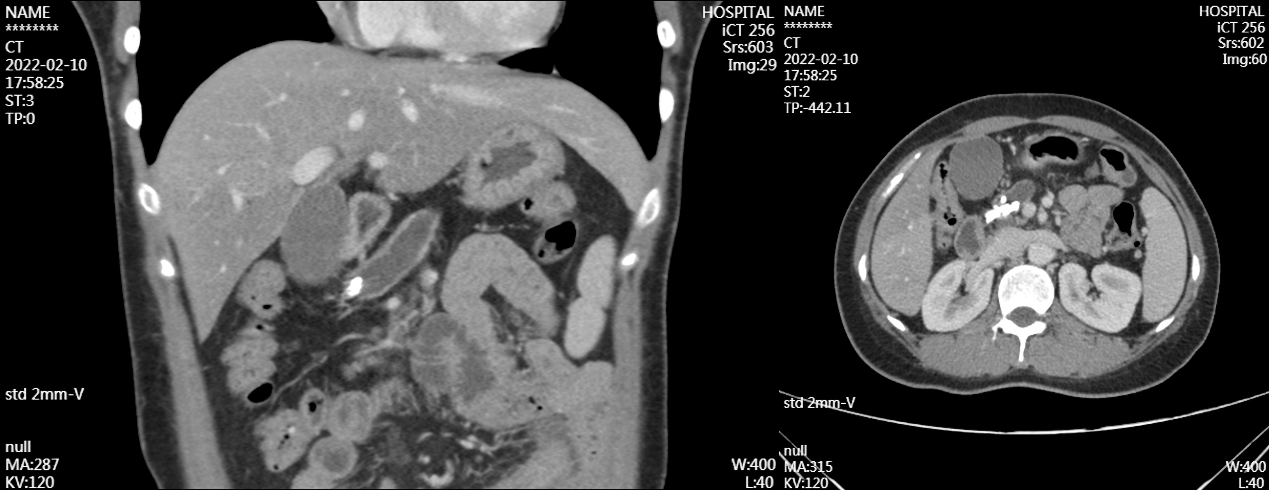


**Figure** **1**


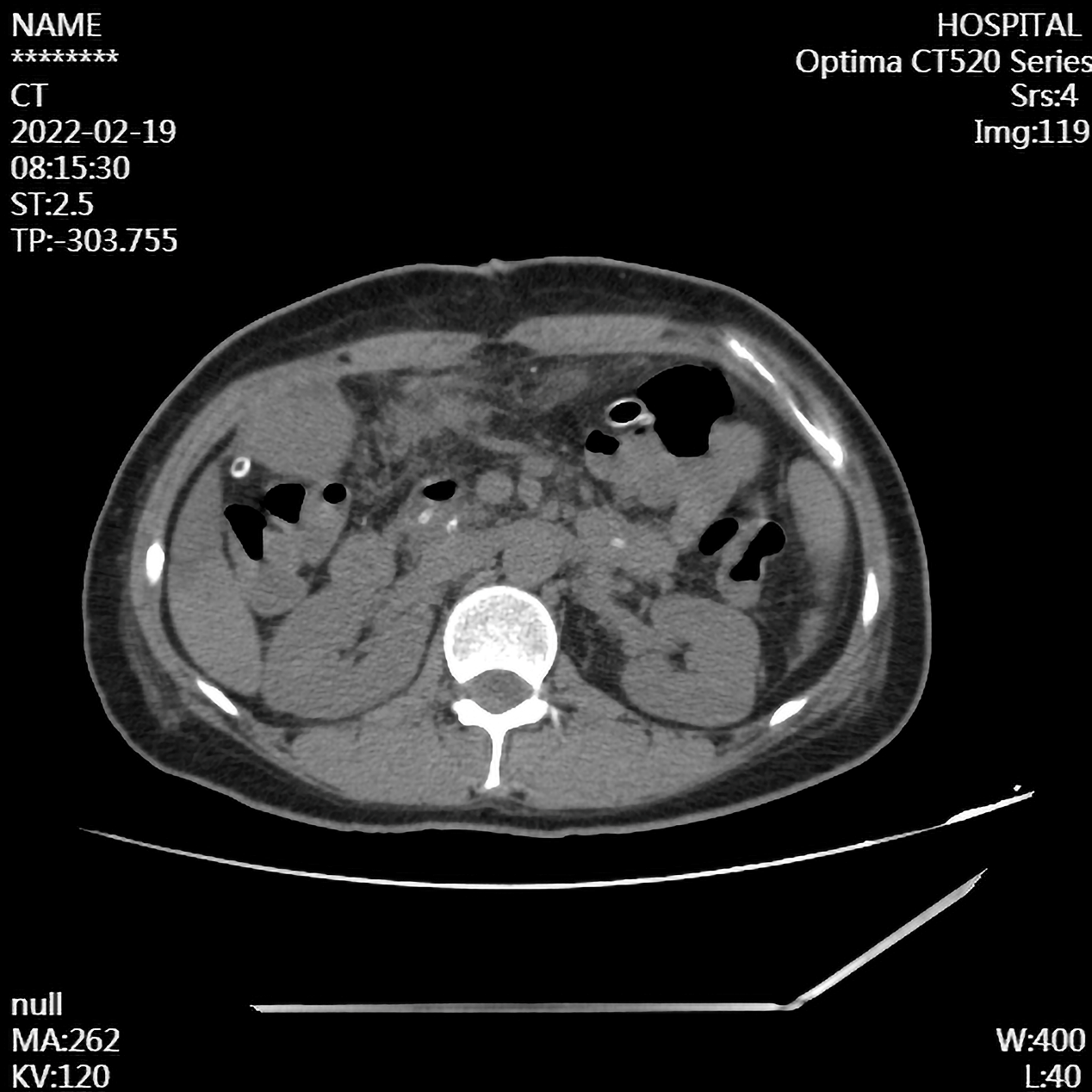


**Figure** **2**


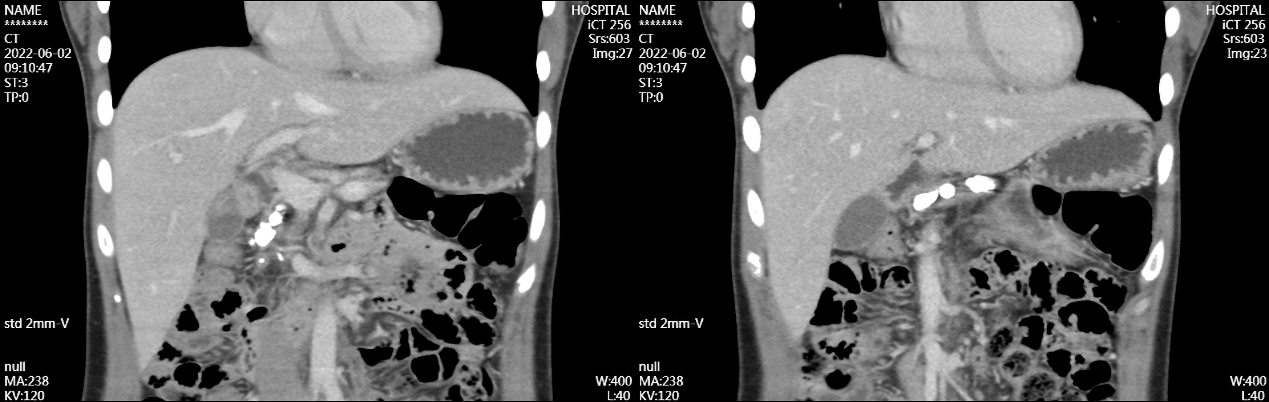


**Figure** **3**


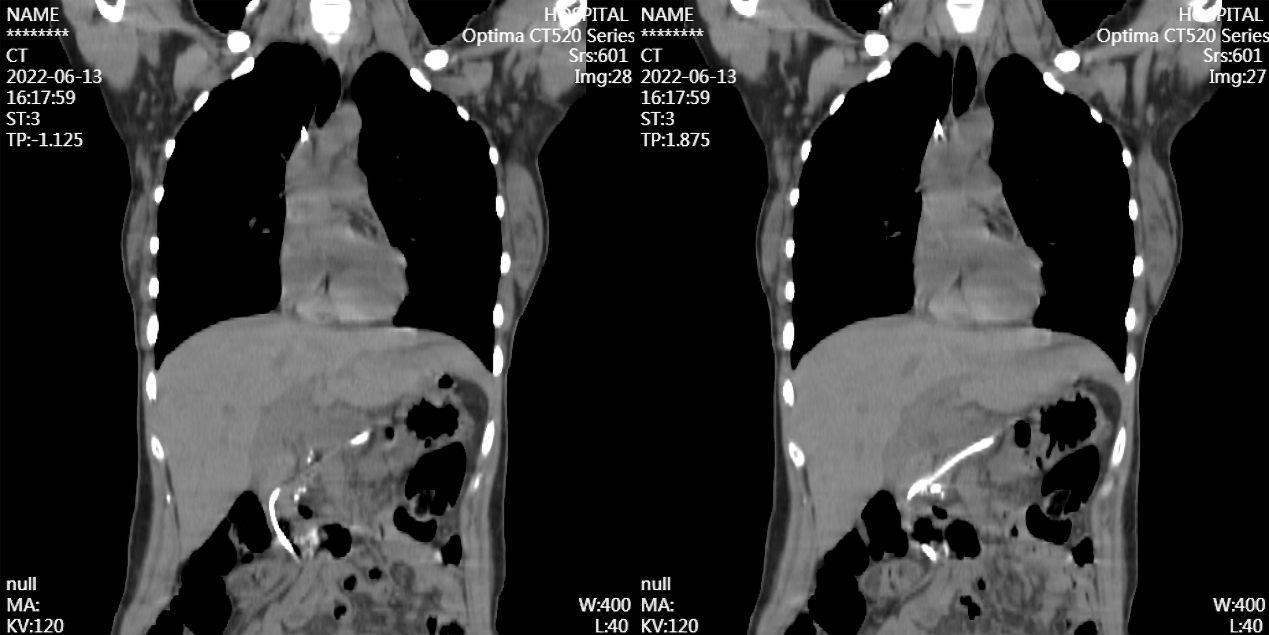


**Figure** **4**
